# Inconsistency in the expression pattern of a five-lncRNA signature as a potential diagnostic biomarker for gastric cancer patients in bioinformatics and *in vitro*

**DOI:** 10.22038/IJBMS.2022.62181.13762

**Published:** 2022-06

**Authors:** Mahmoud Ghanei, Arash Poursheikhani, Azadeh Aarabi, Negin Taghechian, Mohammad Reza Abbaszadegan

**Affiliations:** 1 Medical Genetics and Molecular Medicine Department, Medical School, Mashhad University of Medical Sciences, Mashhad, Iran; 2 Medical Genetics Research Center, Medical School, Mashhad University of Medical Sciences, Mashhad, Iran; 3 Human Genetics Division, Immunology Research Center, Avicenna Research Institute, Mashhad University of Medical Sciences, Mashhad, Iran; 4 Department of Chemistry, Faculty of Science, Ferdowsi University of Mashhad, Mashhad, Iran

**Keywords:** Biomarkers, Long noncoding, RNA, Stomach neoplasms, Transcriptome, Whole exome sequencing

## Abstract

**Objective(s):**

Due to diagnosis of gastric cancer in advanced stages as well as its poor prognosis, finding biomarkers is essential. In this study, using the TCGA RNAseq data of gastric cancer patients, we evaluated the diagnostic value of lncRNAs that had differential expression.

**Materials and Methods:**

We evaluated *P*-value, FDR, and log fold change for whole transcripts. Next, by comparison of the RNAseq gene names with the total known lncRNA names, we identified differential expressed lncRNAs. Following this, specificity and sensitivity for lncRNAs coming from the previous step were calculated. For more confirmation, we predicted target genes and performed GO and KEGG signaling pathway analysis. In the end, we examined reliability and consistency of expression of this signature in three gastric cancer cell lines and one of them in twenty tumors and tumor-adjacent normal tissue samples using qRT-PCR.

**Results:**

Five lncRNAs had proper sensitivity and specificity and had target genes involved in cancer-related signaling pathways; however, they showed different expression patterns in TCGA data and *in vitro*.

**Conclusion:**

The results of our study demonstrated that the five-lncRNAs *PART1, UCA1, DIRC3, HOTAIR*, and *HOXA11AS* require more investigation to be confirmed as diagnostic biomarkers in gastric cancer.

## Introduction

Gastric cancer (GC) is one of the most important leading causes of cancer-related death worldwide (1). According to the report by Global Cancer Observatory (GLOBOCAN) in 2018, GC is fifth in incidence and third in mortality among cancers (both sexes and all ages) across the world, but it is the second and the firth in incidence and mortality in Iran, respectively (2). Despite advancements in the treatment of GC patients based on surgical approaches and targeted drug chemotherapy, poor prognosis and late manifestation of symptoms act as obstacles to early diagnosis of these patients; therefore, identifying patients at advanced stages of the disease can leave most patients untreated (3, 4). Early detection of patients, prediction of outcomes of treatments effectively, identification of new therapeutic targets, and a better understanding of tumorigenesis and progression processes are crucial keys to improving the survival rate of GC patients. Therefore, discovery and development of prognostic and diagnostic biomarkers are essential for the facilitation of early diagnosis, and effective prediction of prognosis, resulting in better outcomes in GC patients.

Long noncoding RNAs (lncRNAs) are attributed as a class of non-coding RNAs (ncRNAs) of greater than 200 nucleotides in length, which have a specific expression in various tissues and diseases such as cancers (5–9). LncRNAs are involved in different biological processes including cell development and differentiation, cell cycle arrest, apoptosis, autophagy, cell senescence, chromosome remodeling, X chromosome inactivation, alternative splicing, RNA decay, embryonic stem cells, cancer cell metastasis, drug resistance, etc. (7, 10–14). According to some studies, lncRNAs have a more important function than protein-coding genes in translational and clinical oncology (11, 15). On the other hand, a group of these noncoding transcripts acts as a tumor suppressor or oncogene and is dysregulated in various types of cancers (16, 17). Other studies have shown that lncRNAs are correlated with cancer recurrence and poor prognosis, although they have not been as yet fully elucidated in GC (18, 19). Since lncRNAs participate in various processes in cancers and are also easily detected, they can be chosen as valuable biomarkers in the diagnosis and prognosis of cancers (20, 21). Several studies have explored the expression of some lncRNAs in GC and introduced several lncRNAs as biomarkers; however, a few have proposed a lncRNA signature, and certainly, there are further lncRNAs that have to be investigated and can be used as practical biomarkers after passing the laboratory steps (22, 23).

In this study, we attempted to perform a comprehensive study using transcriptomic data analysis of patients with GC that are freely available in the Cancer Genome Atlas database (TCGA) (HTTP:// cancergenome.nih.gov) and to provide a new and effective signature for the diagnosis of GC patients. By conducting data mining in RNAseq data of GC patients and thereafter determination of differentially expressed genes and calculating the AUC of the ROC curve, we identified a five-lncRNA signature (*PART1*, *UCA1*, *DIRC3*, *HOTAIR*, and *HOXA11AS*) as a novel potential biomarker for diagnosis of GC patients. We also examined the expression of this signature in three GC cell lines and one of them in twenty tumors and tumor-adjacent normal tissue samples. 

## Materials and Methods

***RNAseq data mining and finding of differentially expressed LncRNAs***

RNAseq data and clinical characteristics were downloaded from the TCGA database to https://cancergenome.nih.gov/. After the data was received, the primary trimming was done and redundant columns and rows were deleted. The RNAseq dataset (STAD) contained 450 transcriptomes, of which 415 were tumors and 35 were tumor-adjacent normal tissue samples.

The RNAseq data processing was continued using the R program. At first, we performed secondary data trimming by determination of the third data quantile and omission of the transcripts that had read counts of fewer than 25 in three-quarters of the data. Applying this command resulted in the removal of 4639 transcripts from the original data set with 20531 transcripts. Then, by calling the edgeR library, logFC, log CPM, *P*-value, and FDR were calculated for each transcript. In the next step, by applying appropriate filtration (*P*-value < 0.05, FDR < 0.05, 1 < logFC < -1) on these components, the list of genes with differential expression was obtained. This list included all types of transcripts with differential expression, from which lncRNAs were to be identified. For this purpose, a list of all identified lncRNAs was obtained from the HUGO Gene Nomenclature Committee (HGNC) website and aligned with the list of differential expression genes to identify the lncRNAs with differential expression. The result was 14 lncRNAs, which were nominated for the next step of the analysis. 

***ROC curve***

This step was done using SPSS software to determine the sensitivity and specificity of the lncRNAs from the previous step, which ultimately led to selection of 5 lncRNAs as potential diagnostic biomarkers. 

***Target gene prediction and functional enrichment analysis***

The target genes of the five-lncRNA signature were predicted by the usage of two online tools including LncRRIsearch and lncRNA2Target. The sum of the identified target genes for these 5 lncRNAs was 238 by LncRRIsearch, after the removal of duplicates, and 115 by LncRNA2Target. Furthermore, the functional analysis of the GO annotation, DO and KEGG signaling pathways were performed. A *P*-value less than 0.01 was considered statistically significant.

***Protein-protein interaction network***

To predict the relationships between the lncRNAs target gene products in-network, the ID number of the lncRNAs target genes and STRING online tool were used. The highest confidence score (a combined score >0.900) was considered significant. Globe-shaped proteins have been characterized based on their association with other proteins. The target genes with multiple connections to other target genes appear to play important roles in the protein-protein interaction network. 

***Experimental design***

Appropriate experimental design is a necessity for gene expression studies. Since RNA is a sensitive molecule and prone to damage, strict conditions must be applied in dealing with it. The most important difference between the experimental and control groups was in the presence or absence of gastric malignancy. In this study, 3 GC cell lines as experimental group 1 with 20 healthy tissue samples as control group 1, and also 20 tumor tissue samples as experimental group 2 with 20 tumor-adjacent normal tissue samples as control group 2 were compared for definite gene expression. All information about experimental procedures, control groups, replicates, experimental conditions, and methods of working with samples in each group were carefully determined and recorded before starting gene expression studies. The qPCR assay was performed at the Immunology Research Center, Avicenna Research Institute, Mashhad, Iran.

***Samples***

Normal tissue samples as a control group were obtained from 20 non-cancerous individuals that had been scanned for gastroesophageal diseases via upper endoscopy in Imam Reza hospital, Mashhad, Iran. Healthy control tissue samples were confirmed by pathological examination. The fresh specimens were immediately transferred to RNA later solution and then stored at -70 °C before RNA extraction. RNA extraction was normally performed from 40–50 milligrams of tissues until two days after tissue sampling. Written informed consent was obtained from all participants in this study before tissue sampling.

Three GC cell lines, MKN-45, AGS, and EPG were obtained from the National Cell Bank of Iran, Tehran. 5×10^5^ cells from AGS, MKN-45, and EPG cell lines were grown in DMEM-high glucose (Gibco™, Cat. No. 11965092, United States), DMEM-low glucose (Gibco™, Cat. No. 11885084, United States) and RPMI 1640 (Gibco™, Cat. No. 11875093, United States) media containing 10% fetal bovine serum (Gibco™, Cat. No. 26140079, United States), and Penicillin-Streptomycin (Gibco™, Cat. No. 15140122, United States), respectively. For all used cell lines, STR profiling was performed for determination of cell line identity; the report was compared with standard cell lines by the usage of matching criteria based on an algorithm that compares the number of shared alleles between two cell lines, expressed as a percentage. Cell lines with ≥80% match are considered to be related, derived from common ancestry. Cell lines with between 55% and 80% match require further analysis for authentication of relatedness; all three cell lines had an acceptable resemblance. Finally, we extracted RNA from 5×10^5 ^cells from each cell line.

***RNA extraction***

Total RNA was extracted with RNX-Plus solution (SinaClon, Cat. No. RN7713C, Iran), chloroform (Merck™, Cat. No. 102445, Germany), 2-propanol (Merck™, Cat. No. 109634, Germany), and 75% ethanol (Merck™, Cat. No. 100983, Germany) according to the common protocol used in molecular laboratories. Next, the concentration and purity of the extracted RNAs were evaluated by Spectrophotometer, Biwave II (Biochrom, UK) which had a concentration and 260/280 ratio of 170–415 ng/µl and 2-2.1, respectively. We also used 2% Agarose gel electrophoresis to ensure RNA integrity by seeing the 18s and 28s bands related to rRNA. Extracted RNAs dissolved in DEPC water were stored at -70 °C until used for cDNA synthesis. For eliminating probable DNA pollution and just before starting cDNA synthesis, we treated the extracted RNAs with DNase1 (Thermo Fisher Scientific, Cat. No. EN0521) according to the manufacturer’s protocol (Table 1).

***Reverse transcriptase***

Reverse transcription reactions were performed in two steps using the NG dART RT kit (EURx, Cat. No. E0801-03) according to the manufacturer’s protocol. The reactions were done in a total volume of 20 µl, detailed in Table 2. The Oligo dT and Random Hexamer primers were used in the cDNA synthesis process at a concentration of 10 picomolar (ρM). Oligo dT is only used to convert transcripts with poly A tail and Random Hexamer to convert transcripts with or without poly A tail to cDNA. The reverse transcriptase (RT) prepared in this kit is an M-MLV (Moloney murine leukemia virus) RT that is suitable for reverse transcription of long transcripts such as lncRNAs; this enzyme was provided at a concentration of 200 U/µl. After the reaction, the synthesized cDNAs were stored at -20 °C. 

***qPCR target information***

The proposed diagnostic signature of this study included five lncRNAs prostate androgen-regulated transcript 1 (*PART1*, NR_028509.1), urothelial cancer-associated 1 (*UCA1*, NR_015379.3), disrupted in renal carcinoma 3 (*DIRC3*, NR_026597.2), HOX transcript antisense RNA (*HOTAIR*, NR_003716.3) and HOXA11 antisense RNA (*HOXA11AS, *NR_002795.2). *PART1, UCA1, DIRC3, HOTAIR,* and *HOXA11AS* have 5914, 2314, 3910, 2364, and 1628 base pairs (bp) of lengths, respectively. Except for *PART1* and *HOTAIR*, which have three splice variants, the rest of the non-coding RNAs have only one variant. The product sizes of qPCR for the lncRNAs were 181, 151, 73, 175, and 89 bp for PART1, UCA1, DIRC3, HOTAIR, and HOXA11AS, respectively. The designed primers were assessed using NCBI Primer-BLAST and showed 100% specificity and acceptable secondary structure scores. These primers were designed to amplify part of exon 1 of PART1, exon 3 of UCA1, exon 1 of DIRC3, exon 7 of HOTAIR, and exon 1 and 2 of HOXA11AS. 

***qPCR Primers***

The reference sequence of the lncRNAs PART1 (NR_028509.1), UCA1 (NR_015379.3), DIRC3 (NR_026597.2), HOTAIR (NR_003716.3), and HOXA11AS (NR_002795.2) was achieved from the NCBI gene database. The primer design was performed using the primer3 online tool, and specificity and secondary structure formation of the primers were checked by NCBI Primer-BLAST. All primers used in this study were synthesized by Pishgam Biotechnology Company and purified by the HPLC method. The sequences of primers used along with their PCR product length are shown in Table 3.

***qPCR protocol***

qPCR analysis of the five lncRNAs was performed on a Light Cycler 96 (Roche Life Science, Roche Diagnostics GmbH, Germany) using the RealQ Plus 2X Master Mix Green (AMPLIQON, Cat. No. A323402, Germany), which is composed of TEMPase Hot Start DNA Polymerase, dNTPs, fluorescent dye and an optimized buffer system. When the fluorescent green dye is free in the solution, it emits a very low fluorescent signal. As soon as the dye binds to the double-stranded DNA, the signal increases significantly (thousandfold), which makes the fluorescent signal of the dye directly proportional to the amount of amplified dsDNA. In this study, 40 temperature cycles were used for amplification of the targets. The amount of materials used for a 0.2 ml qPCR tube stripe in each reaction is shown in Table 4. The temperature program defined for the Real-time PCR instrument is also described in Table 5. All reactions were conducted in duplicate form, and the expression of the five lncRNAs was normalized to the expression level of the housekeeping gene *GAPDH*. 

***qPCR validation***

We optimized the qPCR reactions by the use of the Labcycler Gradient machine (SensoQuest GmbH, Germany). Negative control was used to ensure the absence of contamination and the accuracy of qPCR reactions. Also, to determine the specificity of the amplified products, a melting curve was drawn for all reactions after the completion of the reaction cycles. Each product or amplicon, depending on its specific Tm, will create a unique peak in the melting curve. The primer dimer also creates a peak in the melting curve, which due to its small size and low Tm is below 80 °C, and thus in most cases will be separable from the desired PCR product. In cases where the melting curve was not a single peak or was different from the expected values, the qPCR product was also run on 2% agarose gel. If non-specific bands are seen on the agarose gel, these bands can be partially removed by changing the PCR reaction parameters. For example, by reducing the amount of reaction primers, the band observed in the primer dimer range can be removed, or non-specific bands that have a long length can be removed by reducing the extension phase time or increasing the temperature of this phase.

***Data analysis***

ROC curve analysis was performed using IBM SPSS Statistics v. 25 to determine the sensitivity and specificity of potential biomarkers. An AUC greater than 0.7 was considered a cut-off. Changes in gene expression in tumor tissues or cell lines compared with normal tissues were evaluated using ∆∆CT calculation and Log Fold Change determination. For statistical analysis of real-time results, the ANOVA test was used for cell lines and paired t-test was used for tissue samples, which was done using SPSS software. A *P*-value<0.05 was considered statistically significant. 

## Results

***Differentially expressed Genes***

The data demonstrated that 3722 genes (1840 up-regulated and 1882 down-regulated) were differentially expressed in STAD. Moreover, 14 lncRNAs (139 up-regulated and 49 down-regulated) were identified that were most deferentially expressed in patients. The data are presented in Tables 6 and 7. 

***ROC curve indicates good performance for the suggested diagnostic signature***

**Figure 1:**
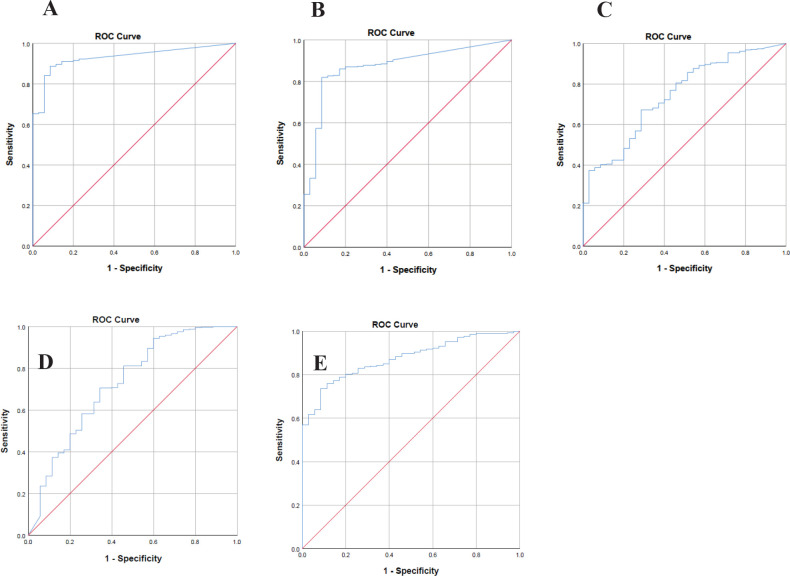
ROC curve analysis of the lncRNAs. A. *HOTAIR*, B. *HOXA11AS*, C. *UCA1,* D. *DIRC3*, E. *PART1*. Area under curve (AUC) was used to determine the sensitivity and specificity of biomarkers. Cut-off was considered 0.7

**Figure 2:**
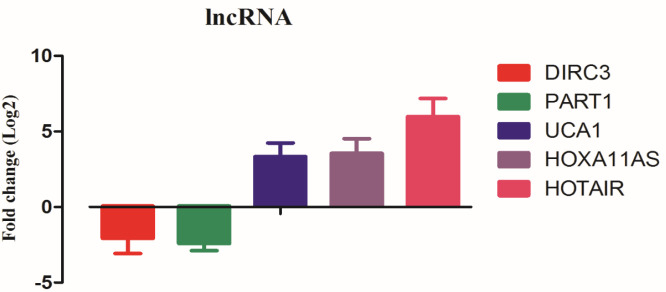
Expression pattern of the suggested lncRNA signature in the TCGA RNAseq data (STAD project)

For evaluation of the diagnostic power of the fourteen-lncRNAs achieved from the RNAseq data analysis by the R program, we calculated the AUC of the ROC curve. A higher AUC represents a better performance and an AUC of more than 0.7 is acceptable and is considered good efficiency. A *P*-value less than 0.05 was considered a significant level. According to AUC and *P*-value, eventually, five lncRNAs (*PART1, UCA1, DIRC3, HOTAIR,* and *HOXA11AS*) were selected as the candidate diagnostic signature. These selected lncRNAs had good potential sensitivity and specificity for diagnosis of GC patients (Figure 1). The expression pattern of these lncRNAs are shown in Figure 2.

***The target gene prediction***

The target genes of the diagnostic lncRNAs signature were predicted by LncRRIsearch (24) and LncRNA2Target (25) online tools. The number of the target genes identified for lncRNAs PART1, UCA1, DIRC3, HOTAIR, and HOXA11AS by the LncRRIsearch database were 100, 100, 33, 100, and 100, respectively (this database represents the top 100 genes identified for the lowest sum of energy) and by the lncRNA2Target database were 0, 55, 0, 68, and 10, respectively. In total, 433 target genes from the LncRRIsearch database and 133 target genes from the LncRNA2Target database were identified for this signature. After deleting duplicates and merging two lists, a list with 353 target genes was generated. 

***GO annotation, disease ontology, and KEGG signaling pathways***

**Figure 3:**
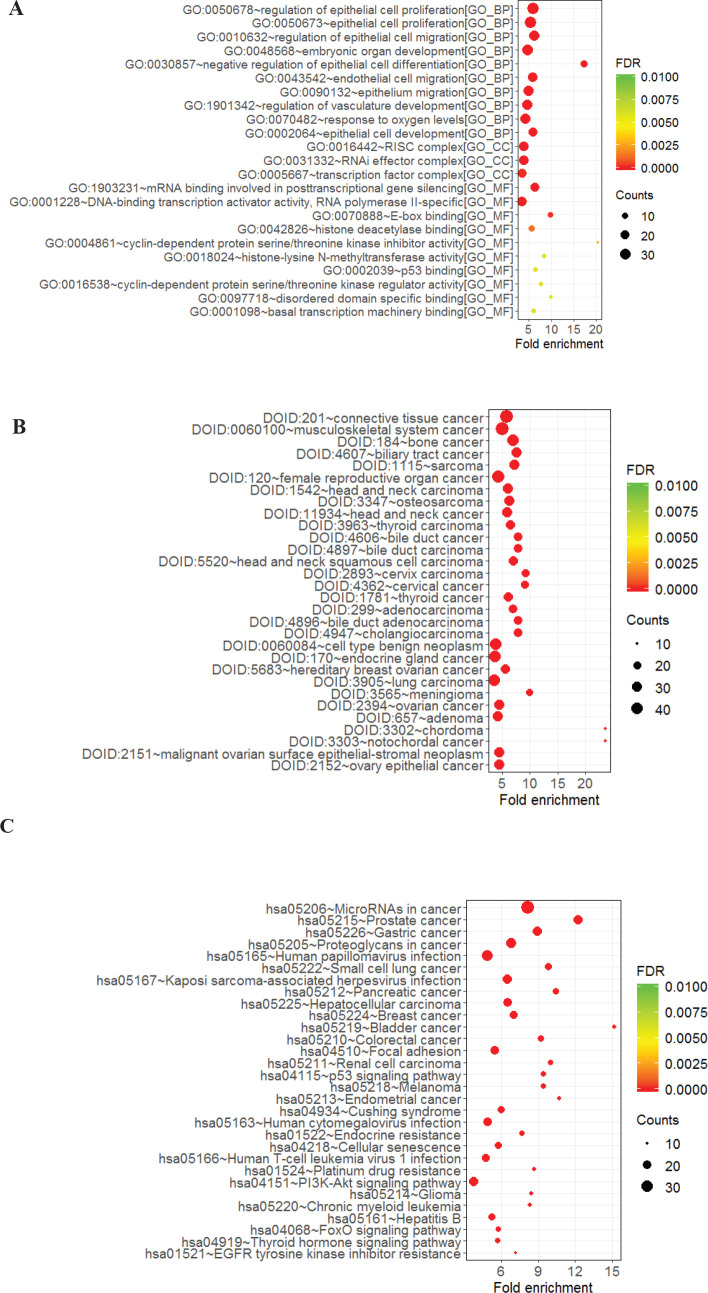
GO, DO, and KEGG pathway enrichment analysis of the differentially expressed genes (Top 20 GO enrichment are presented). A. GO, B. DO, and C. KEGG pathway

For more investigation of the potential biological function and the mechanism of the suggested diagnostic signature, we took advantage of GO annotation, disease ontology, and KEGG signaling pathway analysis for 353 target genes from and disease ontology (DO) indicate activation of cancer-related pathways such as GC, prostate cancer, small cell lung cancer, pancreatic cancer, breast cancer, bladder cancer, colorectal cancer, melanoma, glioma, connective tissue cancer, bone cancer, renal carcinoma, etc. (Figure 3).

***Functional predictions and PPI network construction***

**Figure 4:**
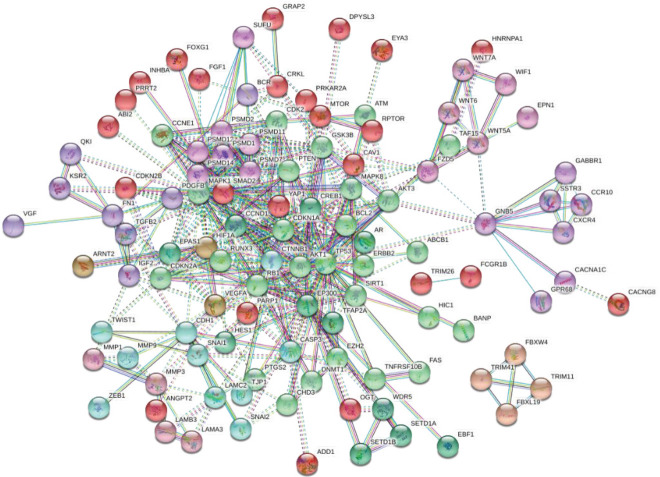
Protein-protein interaction (PPI) network of the differentially expressed genes of STAD (score > 0.9000)

To predict and visualize the protein-protein interactions among the target genes of the five-lncRNA signature, we used the STRING (26) software. For this purpose, to obtain protein-protein interaction (PPI) data, the target genes were first introduced on the STRING website. Next, the cut-off greater than 0.900 for a combined score of PPIs was considered an appropriate criterion for selection and construction of PPI networks. In this network, unconnected nodes are not displayed. Of the 353 target genes identified, 252 genes were involved in constructing nodes of this network (Figure 4). The genes with connections/interactions more than 7 (degree > 7) were filtered. STRING data demonstrated that these lncRNAs contributed to several canonical signaling pathways related to proteins such as cyclins and related *CDKs, TWIST1, CDH1, MMPs, TP53, RB1, WNT7A, GSK3B, ATM, PTEN, BCR, ERBB2, VEGFR, SMAD, TGFB2, MAPK1, FGF1, AKT1,* and *HIF1A*.

***Functional analysis via co-expressed target genes***

At the beginning of this study and before selecting the appropriate lncRNAs as biomarkers, a list of genes with differential expression of TCGA data was obtained and then a list of target genes was predicted by online tools for this signature. To probe the functional value of the signature in GC (stomach adenocarcinoma), we searched the predicted target genes that were co-expressed with our suggested signature and we found a list of 212 genes with this characteristic. In this new list, there are many master genes such as *CDKs, TWIST1, CDH1, MMPs, TP53, RB1, WNT7A, GSK3B, ATM, PTEN, BCR, ERBB2, VEGFR, SMAD, TGFB2, MAPK1, FGF1, AKT1,* and *HIF1A* that were involved in cancer-related pathways and therefore, dysregulated in many cancers. 

***Real-time PCR verification***

**Figure 5:**
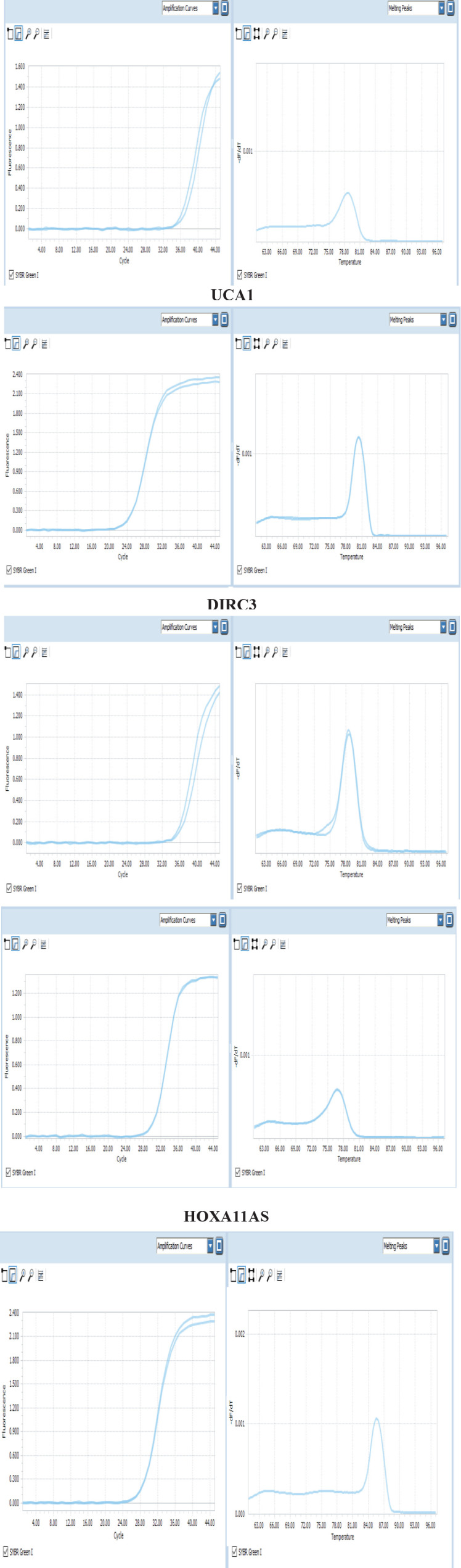
Examples of amplification and melting curves for the lncRNAs PART1, UCA1, DIRC3, HOTAIR, and HOXA11AS in the cell lines and tissue samples

We investigated the expression level of the suggested five-lncRNA signature in the three GC cell lines and 20 healthy tissue samples (Examples of amplification and melting curves for the five lncRNAs are shown in Figure 5). We could not confirm a similar expression pattern for these lncRNAs in the cell lines and TCGA RNAseq data of GC patients using real-time PCR. We used paired t-test as a proper statistical analysis to compare expression levels of five-lncRNA signature in three GC cell lines with 20 healthy tissue samples. We found that *PART1* and *DIRC3* had no detectable expression in any of the three cell lines and normal tissue samples. On the other hand, *UCA1* was expressed in whole samples. Two lncRNAs, *HOTAIR* and *HOXA11AS*, were similarly expressed only in the EPG cell line and normal tissue samples. 

We also examined the expression level of *UCA1* in 20 tumor and tumor-adjacent normal tissue samples with confirmed expression levels greater than 2 (logFC > 2) for *TWIST1*. This study showed that there is no significant correlation between the expression level of *UCA1* and *TWIST1* in tissue samples which is in contrast to our expectations for *UCA1* expression. 

**Table 1:** Stages of RNA treatment by DNase 1 for eliminating probable DNA pollution

Quantity	Components
5 μl	RNA (50–500 ng)
1 μl	10X Reaction Buffer with MgCl2
1 unit	DNase I, RNase-free
1 μl	Ribolock
To 12 μl	DEPC
reaction tubes were incubated at 37 °C for 30 min	
1 μl	50 mM EDTA
reaction tubes were incubated at 65 °C for 10 min	

**Table 2:** Stages of cDNA synthesis by EURx kit for preparing template of qPCR reaction

Quantity	Concentration	Components
13 μl	0.1–5 µg	Total RNA
4 μl	5X	5x NG cDNA buffer
1 μl	10 ρM	Oligo dT Primer
1 μl	10 ρM	Random Hexamer primer
1 μl	200 U/ µL	NG dART RT Mix
-	-	DEPC water, nuclease-free
total volume 20 μl		

**Table 3:** Primers designed to study the expression of lncRNAs for qPCR reaction

Product size	Annealing temperature	Reverse primer	Forward primer	Gene
73	60	CCCTACTGTCCTGGTGGAGA	CTCATCTGTCCGACGAAGCA	DIRC3
175	60	GGCTAGGGCTGGTTTCACTT	GGAAGCGAAGGGGTTGTGTA	HOTAIR
89	60	CTCAGTCGGGTCTTTCCCAG	TTTAGAGGCGCTGACATCCG	HOXA11AS
181	60	TGTCCTTTTCCCCTCCGACA	TCCAGAGCCAGCCAATCACT	PART1
151	60	CCCTGTTGCTAAGCCGATGA	GCCAGCCTCAGCTTAATCCA	UCA1
101	60	GTCATTGATGGCAACAATATCCACT	GGAAGGTGAAGGTCGGAGTCA	GAPDH

**Table 4:** Preparation of qPCR reaction mix for evaluating the expression level of lncRNAs

Components	Concentration	Quantity
**cDNA**	-	2 μl
**RealQ Plus 2X Master Mix Green**	2X	10 μl
**F+R Primers**	10 ρM	1 μl
**Distilled Water**		7 μl
Total **volume** 20 μl		

**Table 5:** Temperature program defined for Real-time PCR instrument for amplifying interest regions of lncRNAs

Process	Temperature °C	Time	Number of cycles
**Hold**	95	10'	1
**Denaturation**	95	5"	40
**Annealing**	60	30"	
**Extension**	72	30"	
**Melting Curve analysis**	70-95	Rising by 0.5°C	1

**Table 6:** Top 20 up and down-regulated genes

Top 20 up-regulated genes				
	**logFC**	**logCPM**	***P*** **-value**	**FDR**
***ENPP7***	-7.24658	2.654383	4.6E-114	7.3E-110
***SLC2A7***	-6.4681	-2.81146	1.04E-89	2.37E-86
***S100G***	-6.41206	0.514534	4.73E-56	2.21E-53
***FLG***	-6.36187	3.289874	5.1E-111	4.1E-107
***GIP***	-6.23295	1.270187	1.89E-38	3.8E-36
***CLDN22***	-6.1317	-3.15007	2.71E-19	5.83E-18
***KRT1***	-6.10628	1.746563	6.56E-80	7.45E-77
***MEP1B***	-6.04667	3.490235	2.25E-65	1.79E-62
***ZG16***	-6.00036	2.506113	1.57E-60	9.99E-58
***AQP10***	-5.97356	2.003138	7.84E-74	7.33E-71
***SLC28A1***	-5.88603	0.267127	3.46E-85	6.88E-82
***ATP4B***	-5.85136	5.777434	5.54E-37	9.89E-35
***G6PC***	-5.65078	0.41213	5.26E-43	1.39E-40
***ATP4A***	-5.57913	5.920505	1.5E-27	9.08E-26
***CRNN***	-5.52021	8.116779	4.7E-13	4.38E-12
***CRCT1***	-5.49336	4.642107	9.88E-21	2.54E-19
***CA7***	-5.47359	-0.32813	2.15E-98	6.83E-95
***ACER1***	-5.3863	-0.21548	2.1E-32	2.34E-30
***KPRP***	-5.35025	1.760696	1.46E-18	2.87E-17
***GYS2***	-5.29783	0.208704	1.46E-43	3.94E-41
Top 20 Down-regulated genes				
	**logFC**	**logCPM**	***P*** **-value**	**FDR**
***ENPP7***	-7.24658	2.654383	4.6E-114	7.3E-110
***SLC2A7***	-6.4681	-2.81146	1.04E-89	2.37E-86
***S100G***	-6.41206	0.514534	4.73E-56	2.21E-53
***FLG***	-6.36187	3.289874	5.1E-111	4.1E-107
***GIP***	-6.23295	1.270187	1.89E-38	3.8E-36
***CLDN22***	-6.1317	-3.15007	2.71E-19	5.83E-18
***KRT1***	-6.10628	1.746563	6.56E-80	7.45E-77
***MEP1B***	-6.04667	3.490235	2.25E-65	1.79E-62
***ZG16***	-6.00036	2.506113	1.57E-60	9.99E-58
***AQP10***	-5.97356	2.003138	7.84E-74	7.33E-71
***SLC28A1***	-5.88603	0.267127	3.46E-85	6.88E-82
***ATP4B***	-5.85136	5.777434	5.54E-37	9.89E-35
***G6PC***	-5.65078	0.41213	5.26E-43	1.39E-40
***ATP4A***	-5.57913	5.920505	1.5E-27	9.08E-26
***CRNN***	-5.52021	8.116779	4.7E-13	4.38E-12
***CRCT1***	-5.49336	4.642107	9.88E-21	2.54E-19
***CA7***	-5.47359	-0.32813	2.15E-98	6.83E-95
***ACER1***	-5.3863	-0.21548	2.1E-32	2.34E-30
***KPRP***	-5.35025	1.760696	1.46E-18	2.87E-17
***GYS2***	-5.29783	0.208704	1.46E-43	3.94E-41

**Table 7:** Differentially expressed lncRNAs for evaluation of diagnostic power by ROC curve

**LncRNAs**				
	**logFC**	**logCPM**	***P*** **-value**	**FDR**
***BCAR4***	5.410524	-0.55488	5.09E-06	1.76E-05
***CDKN2BAS***	-2.45409	0.65801	2.32E-19	5.05E-18
***DIRC3***	-2.05778	-1.47014	2.71E-18	5.12E-17
***DLX6AS***	3.012512	-0.69193	1.99E-06	7.39E-06
***DSCR4***	6.162059	-1.17853	2.5E-06	9.13E-06
***FAM27B***	2.800521	-2.89405	0.029757	0.048477
***CDH19***	-2.7026	1.429299	6.03E-15	7.21E-14
***HOTAIR***	5.983826	1.520031	1.99E-22	6.36E-21
***HOXA11AS***	3.541999	1.706383	1.93E-10	1.26E-09
***HULC***	6.653741	-0.35465	2.83E-07	1.19E-06
***IGF2AS***	3.058415	-1.21374	5.47E-06	1.88E-05
***PART1***	-2.41557	2.244247	9.07E-17	1.38E-15
***TTTY14***	-2.1316	-1.34436	2.58E-07	1.09E-06
***UCA1***	3.323226	4.911589	3.04E-08	1.47E-07

## Discussion

In our investigation, we used the TCGA RNAseq data to introduce a lncRNA signature that can be utilized as a diagnostic biomarker. Our bioinformatics data illustrated that lncRNAs *PART1*, *UCA1*, *DIRC3*, *HOTAIR,* and *HOXA11AS* have more differential expression in the tumor tissues versus normal counterpart margins. Moreover, ROC curve analysis showed that these lncRNAs have significant sensitivity and specificity (diagnostic) values. Furthermore, we investigated the role of the lncRNAs through enrichments and real-time PCR.

It has been demonstrated that down-regulation of lncRNA PART1 blocks cell proliferation and promotes apoptosis in bladder cancer (27). Furthermore, exosome-mediated transfer of lncRNA PART1 can induce chemotherapy resistance in ESCC by competing for endogenous miRNA. PART1 promotes tumorigenesis by miR-143 in colorectal cancer(28, 29). Zhang XQ *et al*. declared that a long non-coding RNA signature can predict the prognosis of the glioblastoma multiform patients (30). 

Overexpression of lncRNA *UCA1* has been suggested to increase cell growth and chemo-resistance by inhibiting *miR-513a-5p* in retinoblastoma cells (31). In addition, *UCA1* overexpression is associated with a poor survival rate in patients with digestive system malignancies (32).  *Shan L et al.* demonstrated that high expression of serum *UCA1* can be considered a potential biomarker for clinical diagnosis of GC (33). It has been suggested that *UCA1* confers endocrine-therapy resistance through EZH2/p21 axis and the PI3K/AKT signaling pathway in breast cancer (34). *UCA1* can promote carcinogenesis by the Wnt signaling pathway in papillary thyroid carcinoma (35). Down-regulation of *UCA1* has been shown to enhance radiosensitivity and inhibit cell migration by suppressing EMT in colorectal cancer cells (36). Moreover, *Wang J et al.* found that knockdown of *UCA1* increases cisplatin sensitivity in tongue squamous cell carcinoma cells (37). 

Previous investigations have reported that lncRNA *DIRC3* expression had an impact on carcinogenesis. This lncRNA has been regulated by *MITF-SOX10* in melanoma tumors (38). *Shen Z et al.* showed that *DIRC3* and near *NABP1* genetic polymorphisms are associated with poor prognosis in laryngeal squamous cell carcinoma patients (39)60.75+/-10.082. 

LncRNA *HOTAIR* has been demonstrated to serve as a prognostic and diagnostic biomarker in cancers. It promotes tumor progression via sponging the *miR-217-GPC5* axis in GC (40). *HOTAIR* mediates the switching of histone H3 lysine 27 acetylation to methylation, to promote the EMT process in GC (41). Moreover, it has been reported that *HOTAIR* rs17720428 SNP is correlated with risk and prognosis of GC in the Chinese Han population. *HOTAIR* modulates *KLF12* to regulate GC progression via the PI3K/ATK signaling pathway by sponging *miR-618* (42). It has been reported that lncRNAs such as *H19*, *HOTAIR*, *UCA1,* and *PVT1* could serve as potential diagnostic and prognostic biomarkers in patients with GC (43). *Xiao J et al.* demonstrated that high *HOTAIR* expression promotes proliferation and metastasis in GC via the *miR-126/CXCR4* axis (44).

*Zhao X et al.* exhibited that lncRNA *HOXA11-AS* acted as a ceRNA to promote cisplatin resistance of human LUAD cells via the *miR-454-3p/Stat3* axis (45). *Li N et al.* showed that increased expression of *HOXA11-AS* is a risk factor for poor clinical outcomes in numerous tumors and may act as a novel biomarker for poor prognosis and metastasis in cancers (46). *Su J-C et al.* demonstrated the oncogenic role of *HOXA11AS* in breast cancer, providing novel clues for the future clinical diagnosis and treatment of early-stage breast cancer patients (47). Chen J-H *et al*. showed that up-regulation of lncRNA HOXA11-AS predicted a poor prognosis and lncRNA HOXA11-AS promoted cell epithelial-mesenchymal transition (EMT) by inhibiting miR-200b expression in NSCLC (48). *Liu Z et al. *declared that *HOXA11-AS* not only could promote GC cell migration and invasion *in vitro*, but also promotes GC cell metastasis *in vivo*, at least in part, by regulating *β-catenin* and *KLF2 *(49). LncRNA *HOXA11-AS* was shown to have the ability to distinguish CRC tissue from non-cancerous tissue, and CRC tissue with lymph node metastasis from CRC without lymph node metastasis (50).

To clarify the molecular function of this suggested five-lncRNA signature, we predicted the target genes and the corresponding pathways using GO annotation, DO, and KEGG signaling pathway analysis. The results showed the participation of this signature in essential biological processes such as cell division, transcription regulation, change in expression of growth factors, and enriched KEGG pathways including PI3k-Akt signaling pathways, p53 signaling pathways, and pluripotent stem cell signaling pathways. To further investigate the proposed lncRNA signature, the protein-protein interactions of the target genes were predicted.

The current study indicated inconsistency in the results of real-time PCR in the cell lines and TCGA RNAseq data. LncRNAs *PART1* and *DIRC3* had no detectable expression in any cell lines. These two lncRNAs were down-regulated in tumor TCGA RNAseq samples vs normal margin; lack of expression of them in the cell lines is probably due to very low or no expression of them which emanated from cumulative mutations that cell lines bear in passing time, presence of different subclones in culture, and so their complex and unclear interactions, the different circumstance of cells in culture vs body, and other causes that are still unclear. The function of lncRNAs *PART1* and *DIRC3* is unclear in GC; therefore, future studies should focus on these lncRNAs and clarify their function in GC. On the other hand, lncRNA *UCA1* was expressed in all three cell lines; however, its expression differed from TCGA data. The probable reasons for its expression in all three cell lines could be its more important role in gastric carcinogenesis, more stability, and more expression; however, deviation in the expression pattern with TCGA data is among the causes that are very difficult to comment on. Nevertheless, *HOTAIR* and *HOXA11AS* were expressed just in the EPG cell line and had no detectable expression in the other two cell lines. This can be explained by the role of these lncRNAs in the EPG cell line but in no others, because of the different genetic contexts of the three cell lines. Overall, the exact interpretation of these discrepancies between the results of bioinformatics and laboratory studies requires further study, and here we have only stated a series of hypotheses. 

 Activation of the PI3K-Akt pathway is reported in many malignancies. Inhibition of the PI3K-Akt pathway can induce apoptosis and decrease cell division via negative regulation of *Plk1* both *in vitro* and *in vivo* (51, 52). One of the most important genes involved in this pathway is *TWIST1*. This gene normally is overexpressed in many cancers and promotes metastasis using activation of the EMT process (53). *TWIST1* was predicted as an important target gene for UCA1 and also existed in the differential expression gene list resulting from TCGA data analysis. To examine the correlation between *TWIST1* and *UCA1*, the lncRNA that had been expressed in all three cell lines, we performed real-time PCR for *UCA1* in twenty tumor and tumor-adjacent normal tissue samples with confirmed overexpression of *TWIST1* (logFC > 2). This examination showed that *UCA1* was up-regulated and down-regulated in 8 and 3 samples, respectively. In the other 9 tissue samples, *UCA1* did not show altered expression. Altogether, the evaluation of *UCA1* using paired t-test did not show a significant statistical relationship in twenty tissue samples; this was another important contradiction that we encountered during this study. Discrimination in sampling (selection of samples with high *TWIST1* expression) seems to be one of the reasons for this unexpected result; However, based on bioinformatics analysis, our sampling was correct and the results should have been different. 

Overall, like many other studies, our study had its limitations. First, the data used for this study to introduce a diagnostic signature were extracted from a single database (TCGA); the use of more databases such as GEO certainly makes the results more reliable. Second, due to the use of cell lines in this study for investigation of suggested signature expression and the differences between these cells in terms of growth conditions and cumulative mutations, as well as the low number of cell lines used, it is necessary to examine the expression of these 5 lncRNAs in more tissue samples.

## Conclusion

The bioinformatics analysis of TCGA RNAseq data presented a lncRNA signature that seemed to be useable as a diagnostic biomarker. The proposed lncRNA signature including *PART1*, *UCA1*, *DIRC3*, *HOTAIR,* and *HOXA11AS* showed more differential expression in the TCGA RNAseq data of GC patients. Additionally, drawing ROC curves exhibited them properly as biomarkers. Enrichment analysis confirmed the role of the signature in the critical biological processes and pathways. Furthermore, we predicted the target genes of the signature and protein-protein interactions among them. So far, all surveys have supported the biomarker role of this signature, however, the results of real-time PCR in cell lines and tissue samples were inconsistent with the findings of the analysis of RNAseq TCGA data, which is difficult to fully interpret using the findings of this study. 

We could not conclusively present a five-lncRNA signature with diagnostic potential for GC because of some identified contradictions in bioinformatics and laboratory study. More investigations should be performed for ultimate validation or rejection of this signature: investigations that cover our faults using a large sample size, examination of these results in tissue samples, and also a further exploration of the biological and molecular mechanism of the suggested five-lncRNA signature in GC progression. 

## Authors’ Contributions

GM, PA, and AMR Conceived the study. GM, PA, AMR, AA, and TN Performed interpretation and analysis of data. GM and PA Prepared the manuscript. AMR Revised the manuscript for important intellectual content. AMR provided supervision.

## Funding

This study was funded by The Vice Chancellor for Research at Mashhad University of Medical Sciences (no. 13986). 

## Conflicts of Interest

The authors declare that they have no competing interests.
